# Leprosy Association with Low MASP-2 Levels Generated by *MASP2* Haplotypes and Polymorphisms Flanking MAp19 Exon 5

**DOI:** 10.1371/journal.pone.0069054

**Published:** 2013-07-30

**Authors:** Angelica Beate Winter Boldt, Isabela Goeldner, Ewalda R. S. Stahlke, Steffen Thiel, Jens Christian Jensenius, Iara José Taborda de Messias-Reason

**Affiliations:** 1 Laboratório de Imunopatologia Molecular – Hospital de Clínicas, Universidade Federal do Paraná, Curitiba, BR; 2 Departamento Estadual de Saúde do Paraná, Curitiba, BR; 3 Department of Biomedicine, Aarhus University, Arhus C, Denmark; National Cancer Center, Japan

## Abstract

**Background:**

The gene *MASP2* (mannan-binding lectin (MBL)-associated serine protease 2) encodes two proteins, MASP-2 and MAp19 (MBL-associated protein of 19 kDa), bound in plasma to MBL and ficolins. The binding of MBL/MASP-2 and ficolin/MASP-2 complexes to microorganisms activates the lectin pathway of complement and may increase the ingestion of intracellular pathogens such as *Mycobacterium leprae*.

**Methods:**

We haplotyped 11 *MASP2* polymorphisms with multiplex sequence-specific PCR in 219 Brazilian leprosy patients (131 lepromatous, 29 borderline, 21 tuberculoid, 14 undetermined, 24 unspecified), 405 healthy Brazilians and 291 Danish blood donors with previously determined MASP-2 and MAp19 levels. We also evaluated MASP-2 levels in further 46 leprosy patients and 69 Brazilian controls.

**Results:**

Two polymorphisms flanking exon 5 of *MASP2* were associated with a dominant effect on high MASP-2 levels and an additive effect on low MAp19 levels. Patients presented lower MASP-2 levels (P = 0.0012) than controls. The frequency of the *p.126L* variant, associated with low MASP-2 levels (below 200 ng/mL), was higher in the patients (P = 0.0002, OR = 4.92), as was the frequency of genotypes with *p.126L* (P = 0.00006, OR = 5.96). The *1C2-l [AG] haplotype, which harbors *p.126L* and the deficiency-causing *p.439H* variant, has a dominant effect on the susceptibility to the disease (P = 0.007, OR = 4.15). Genotypes composed of the *2B1-i and/or *2B2A-i haplotypes, both associated with intermediate MASP-2 levels (200–600 ng/mL), were found to be protective against the disease (P = 0.0014, OR = 0.6). Low MASP-2 levels (P = 0.022), as well as corresponding genotypes with *1C2-l and/or *2A2-l but without *1B1-h or *1B2-h, were more frequent in the lepromatous than in other patients (P = 0.008, OR = 8.8).

**Conclusions:**

In contrast with MBL, low MASP-2 levels increase the susceptibility to leprosy in general and to lepromatous leprosy in particular. *MASP2* genotypes and MASP-2 levels might thus be of prognostic value for leprosy progression.

## Introduction

*Mycobacterium leprae* causes leprosy (Hansen’s disease) by invading macrophages and Schwann cells. This leads to progressive and irreversible disabilities associated with heavy social stigma. Leprosy has been eliminated from 119 countries out of 122 since 1985, but Brazil still presents the second highest prevalence rate in the world with 1.52/10,000 inhabitants, and the highest detection rate of new cases with 17.86/100,000 inhabitants [1].

On exposure to *M. leprae*, only a small group of infected individuals develop the disease, which is characterized by a wide spectrum of immunologically-defined clinical manifestations. The lepromatous form is the result of a Th2 response and characterizes the low resistance pole of Hansen disease. The other pole is represented by the tuberculoid form and is defined by a strong Th1 response controlling bacillus dissemination [2]. The innate immune response interacts with the adaptive Th1 and Th2 responses, e.g. by cells secreting different key cytokines and by generating anaphylatoxins through activation of the complement system. The lectin pathway of complement becomes activated when the pattern recognition molecules mannan-binding lectin (MBL) or ficolins recognize foreign structures. This recognition leads to the activation of MBL-associated serine proteases 1 and 2 (MASP-1 and-2). Recently, it was found that MASP-1 is the first enzyme to get activated leading thereafter to the activation of MASP-2 and culminating with the activation of other complement system components [3], [4]. As a result, fragments of complement components deposit on the pathogeńs surface, leading to opsonization and phagocytic ingestion, a mechanism subverted by *M. leprae* to allow intracellular infection [5].

**Figure 1:**
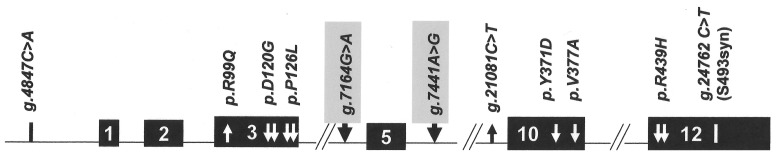
Approximate gene location and association with plasma levels of the investigated *MASP2* polymorphisms. SNPs flanking the alternative exon 5 were investigated for the first time in this study and are gray-shadowed. Arrows pointing downward: SNPs reportedly associated with low MASP-2 levels (400–200 ng/mL) or very low MASP-2 levels (under 200 ng/mL, shown as a double arrow) [9], [10], [12]. Arrows pointing upward: SNPs reportedly associated with high MASP-2 levels (above 600 ng/mL) [12]. SNPs with unknown or neutral effect are indicated without an arrow. Nucleotide substitutions that correspond to the amino acid changes in the NG007289.1 reference sequence are: *g.5557G>A (p.R99Q), g.5620A>G (p.D120G), g.5638C>T (p.P126L), g.21370G>T (p.Y371D), g.21389T>C (p.V377A)* and *g.24599G>A (p.R439H).* Exons are boxed and numbered, intron and exon sizes are not to scale.

Both proteins MASP-2 and MAp19 (MBL-associated protein of 19 kDa, also named small MBL-associated protein or sMAP) are encoded by the *MASP2* gene [6] [7]. MASP-2 and MAp19 share the signal peptide, the first CUB1 domain (an acronym for C1r/C1s, Uegf and bone morphogenetic protein-1 domain) and an EGF domain (epidermal growth factor-like domain). MAp19 has four exclusive C-terminal amino acids, whereas MASP-2 further presents a second CUB domain, two CCP (complement control protein) domains and a serine protease domain [6]. Single nucleotide polymorphisms (SNPs) located in the exons for the CUB1, EGF and serine protease domain have been shown to modify MASP-2 functions [8]–[11]. Other polymorphisms occurring in intron 9 and in exon 10 (encoding the last CCP) were associated with different MASP-2 and MAp19 levels [9], [12], [13] (Figure 1). Based on these SNPs, we recently described and named ten haplotypes according to their phylogenetic relationships [12]. Five haplotypes share *g.24762C* in exon 12 and belong to clade 1: *1A (most ancient), *1B1-h and *1B2-h (both associated with higher MASP-2 levels) and *1C1-l and *1C2-l (who share the *p.126L* amino acid and are associated with low MASP-2 levels – additionally, *1C2-l bears the deficiency-causing *p.439H* variant). Five other haplotypes present *g.24762T* in exon 12 and belong to clade 2: *2A1, *2A2-l (which contains the low MASP-2 levels-associated *p.377A* variant), *2B1-i and the very common *2B2A-i (both associated with intermediate MASP-2 levels), and the MASP-2 deficiency-causing *2B2B-l haplotype (with the *p.120G* variant). *MASP2* polymorphisms and haplotypes have been associated by us with susceptibility to Chagas disease [14] and hepatitis C [13], and by others with bacterial infections after orthotopic liver transplantation [15] and malaria [16]. Most authors investigated only the uncommon *p.D120G* polymorphism and did not find any disease association [17]–[21].

In this work, we investigated if *MASP2* polymorphisms and MASP-2 or MAp19 levels in serum can play a role in the susceptibility to leprosy. We found an association between nine previously described and two formerly not investigated *MASP2* polymorphisms and MASP-2 and MAp19 levels in healthy individuals and in patients with leprosy disease, as well as an association between some of these SNPs and the susceptibility to the disease and to the lepromatous condition.

## Materials and Methods

### Subjects and Samples

Leprosy patients who attended one of two centers in Curitiba in southern Brazil were eligible for the study. Study subjects comprised consecutive outpatients from the Hospital de Clínicas, Federal University of Paraná, State Health Department of Paraná, and inpatients from the Sanitary and Dermatologic Hospital of Paraná. For all 219 patients (37% female, 63% male; 80.4% Euro-Brazilian, 18.7% Afro-Brazilian, 0.9% Amerindian; average age of 50.5 years, range 15–94), leprosy was diagnosed on the basis of the clinical and histopathological features of affected lesions and classified according to the criteria of Ridley and Jopling [22]. The diagnosis at presentation was lepromatous leprosy for 131 (60%), tuberculoid leprosy for 21 (10%), and borderline leprosy for 29 patients (13%); 14 patients (6%) had an undetermined form of leprosy and 24 (11%) were unspecified. Four hundred and five healthy, symptom-free individuals were assessed as control subjects (37.3% female, 62.7% male; 80.7% Euro-Brazilian, 15.3% Afro-Brazilian, 2.2% Amerindian, 1.7% other; average age of 45 years, range 15–89). To reduce the potential for selection bias, we selected controls from different sources: 325 blood donors from three different blood banks in Curitiba (122 from the Hospital de Clínicas – UFPR, 164 from the Hemepar and 64 from the Biobanco of the Hospital Evangélico) and 80 volunteers without leprosy from Curitiba and surrounding cities. Patients and control subjects had the same socioeconomic status and were from the same geographical area. The patients and controls were defined as being from major European or African ascendency based on physical characteristics. Based on HLA allelic frequencies of South Brazilian population samples identified in the same way, Euro-Brazilians have an average sub-Saharan African component of 9% and an average Amerindian component of 5%, whereas Afro-Brazilians have at least 40% of African and 6% of Amerindian ancestry [23], [24]. Furthermore, the genotype distribution of *MBL2* haplotypes of Euro-Brazilians of Southern Brazil, with ascendency defined in the same manner, is homogeneous with the *MBL2* genotype distribution of most European populations, whereas Afro-Brazilians are similar to Mozambicans, resulting from the large eastern African input in Brazil during the first half of the 19th century [25], [26]. All patients and control subjects provided written informed consent. We also obtained written informed consent from the next of kin, caretakers, or guardians on the behalf of the minors/children participants involved. The study was approved by the local medical ethics committee (Comitê de Ética em Pesquisa- Hospital de Clínicas 497.079/2002–06). In order to verify a possible correlation between *MASP2* polymorphisms and protein levels, we also genotyped 291 Danish individuals with known MASP-2 and MAp19 levels, measured with a TRIFMA assay as previously published [27], [28].

### MASP2 Genotyping

We optimized a multiplex sequence-specific amplification method (multiplex PCR-SSP) to rapidly identify nine single nucleotide polymorphisms (SNPs rs61735600, rs7548659, rs72550870, rs56392418, rs17409276, rs12711521, rs2273346, rs12085877 and rs1782455) and ten corresponding haplotypes (*1A, *1B1-h, *1B2-h, *1C1-l, *1C2-l, *2A1, *2A2-l, *2B1-i, *2B2A-i, *2B2B-l) at low cost and with high accuracy, as previously published [12]. We further investigated the SNPs rs2273344 and rs9430347 flanking the alternative exon 5. These SNPs could be responsible for a hitch-hiking effect with the intron 9 *g.21081T* polymorphism, explaining the previous association found by our group with this variant and high MASP-2 as well as low MAp19 levels [12]. To this end, we amplified a 316 bp fragment specific for *NG_007289.1:g.7164G>A* (rs2273344) in intron 4 and *NG_007289.1:g.7441A>G* (rs9430347) in intron 5 using the *MASP2* Intr4_rs2273344Gf (5′ GTTCCCTGCACTGTGGGACG 3′) and *MASP2* Intr4_rs2273344Af (5′ GTTCCCTGCACTGTGGGACA 3′) forward sequence-specific primers (SSPs) conjugated with the *MASP2* Intr5_ rs9430347Ar (5′ CTCCCACCCCAGAGACACGT 3′) and *MASP2* Intr5_rs9430347Gr (5′ CTCCCACCCCAGAGACACGC 3′) reverse SSPs. In order to control for the quality of the reactions, we added two generic primers (*HGH*f: 5′ TGCCTTCCCAACCATTCCCTTA 3′ and *HGH*r: 5′ CCACTCACGGATTTCTGTTGTGTTTC 3′) to amplify a fragment of 431 bp of the human growth hormone gene (*HGH*). The amplification protocol starts with a 3 min denaturation step at 96°C, followed by 30 cycles of 15 sec at 94°C, 30 sec at the specific annealing temperature and 30 sec at 72°C, concluding with 5 min at 72°C in the final DNA extension step. Annealing temperature decreased every 10 cycles (69°C, 67°C and 65°C), according to a previously published “touch-down” strategy which assures higher specificity to the amplification, while providing a larger amount of the desired PCR product [29]. The haplotypes defined by two SNPs, amplified by a pair of SSPs, were identified by the presence or absence of specific bands after agarose gel electrophoresis. Control bands informed on the quality of the reactions.

### MASP-2 Concentration Assay

We measured MASP-2 concentrations in 1∶40 diluted sera of 45 patients and 82 controls with the same proportion of selected *MASP2* genotypes, using the enzyme-linked immunosorbent assay HK326 (Hycult Biotechnology, Uden, The Netherlands).

### Statistics

Genotype, allele and haplotype frequencies were obtained by direct counting. Neighbor SNPs distributed from the promoter to exon 12 were phased with the SSP primers. In most cases, the phase between distantly situated SNPs could be deduced due to strong linkage disequilibrium between the variants. The hypothesis of Hardy–Weinberg equilibrium and of homogeneity between genotype distributions (exact test of population differentiation of Raymond and Rousset) were verified with the ARLEQUIN software package version 3.1 (http://anthro.unige.ch/arlequin/). Tests of independence between patients and controls, as well as between patients with the lepromatous and non-lepromatous forms, were performed using Fisher exact test. Corresponding false discovery rate q-values were calculated with the Microsoft research false discovery rate tool for 2×2 tables. MASP-2 levels were compared between the groups using nonparametric Mann-Whitney/Kruskal–Wallis tests (or ANOVA if data passed normality tests). The statistical analysis was undertaken using the GraphPad Prism 3.0 software package. Two-tailed P-values less than 5% were considered significant. They were additionally corrected with the Bonferroni method (shown as P_Bf_). HAPSTAT software was used to evaluate associations between SNPs or haplotypes and leprosy *per se* as well as lepromatous leprosy, adjusted for age, gender and ethnic group. HAPSTAT software reconstructs haplotypes using an expectation maximization (EM) algorithm to calculate maximum likelihood estimates of haplotype frequencies, while taking into account phase ambiguity. It evaluates association within the additive, recessive, dominant, codominant additive and codominant recessive models [30]. EM-generated haplotype frequencies were somewhat different to those generated by physical PCR-SSP haplotyping, with 2.3–3.4% discrepancy, which is comparable to the percentage previously reported for *MBL2* in the Gabonese population [26]. We choose the best-fitting model between significant additive, recessive, dominant, codominant additive and codominant recessive models adjusted for age, ethnic group and gender distributions, based on the lowest information criterion of Akaike [31]. We also used the default parameters of the Human Splicing Finder program to evaluate in silico possible effects of the intron 4 and 5 variants [32]. Logistic regression models were used to adjust results for age, gender and ethnic group, as well as for previously published *MBL2, FCN1* and *FCN2* genotypes [33]–[35], using STATA v.9.2 (Statacorp, USA).

## Results

### MASP2 Polymorphisms and Haplotypes

Genotype distribution was in Hardy-Weinberg equilibrium. Different combinations of nine out of 11 investigated polymorphisms (*g.4847A>C* in the promoter, *p.R99Q, p.D120G* and *p.P126L* in exon 3, *g.21081C>T* in intron 9, *p.Y371D* and *p.V377A* in exon 10, *p.R439H* and *g.24762T>C* in exon 12) resulted in 11 haplotypes, one of them being a possible recombinant between the promoter-exon 3 block of *2B2A-i and the exon 9– exon 12 block of *1B1-h, thus named *2B2A-i.1B1-h [*NG_007289.1:g.4847A*, *p.99R, p.120D, p.126P×g.21081T, p.371D, p.377V, g.24762C*]. Further six haplotypes presented recombination of the region surrounding the MAp19 alternative exon 5, as defined by the SNPs rs2273344 and rs9430347. The SNPs occurred in three different combinations *in cis*, *AG* being the most common (around 85%), followed by *GA* (around 15%) and *GG*, which appeared only twice in the patient group, clustered in the *2B2A-i haplotype. Despite being rare in the human, *GG* is conserved in the chimpanzee (BAC clone CH251-686E20 from chromosome 1). The phylogenetic nomenclature adopted for *MASP2* cannot accommodate recombinant haplotypes whose parental haplotypes are not clear, thus we decided to add *[AG]*, *[GA]* or *[GG]* to the original names to account for the information regarding these polymorphisms.

### MASP2 Association with MASP-2 and MAp19 Levels

**Figure 2:**
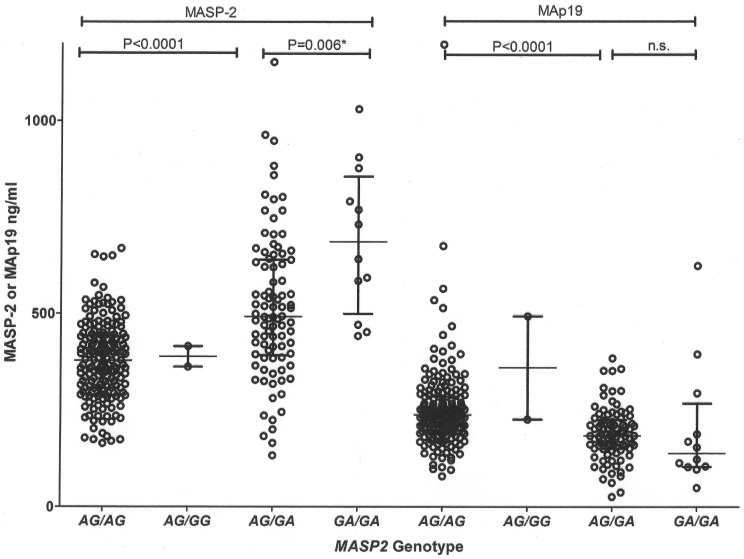
MASP-2 and MAp19 levels in groups of exon 5 flanking *MASP2* genotypes in the Danish population. Data is shown with medians and interquartile ranges. One outlier (1198 ng/mL in the *AG/AG* group) was excluded for better visualization of MAp19 levels. Mann-Whitney P values were calculated between *AG/AG* and *AG/GA*, *AG/GA* and *GA/GA* groups. n.s.: not significant,* P_Bf_ = 0.03.

The *AG* variant combination flanking exon 5 occurred in all haplotypes excepting *1B2-h, which is associated with high MASP-2 levels. On the other hand, *GA* occurred in all haplotypes except in *1C1-l, *1C2-l, *2A2-l and *2B2B-l, which are all known to be associated with low MASP-2 levels [9], [10], [12]. In the Danish population, where we did not find (and thus expect no interference from) the *1B2-h, *1C1-l and *1C2-l haplotypes, *AG/AG* genotypes presented lower MASP-2 levels than *AG/GA* and *GA/GA* genotypes (medians: 378<492<686 ng/mL, respectively, Kruskal-Wallis P<0.0001). The opposite occurred with MAp19 levels: *AG/AG* presented higher values than the other genotypes (medians: 238.1>184.4 and 138.6 ng/mL, respectively, Kruskal-Wallis P<0.0001) (Figure 2). In silico, the *g.7164G* (rs2273344) of the *GA* allelic combination creates the silencer motif 5′-GGGCCA-3′, which could be responsible for skipping the alternative exon 5, increasing MASP-2 and reducing MAp19 levels. The *GA* combination also shares a highly significant, strong dominant effect on generating MASP-2 levels higher than 600 ng/mL with four other minor alleles: *g.4847C* in the promoter (rs7548659), *g.21081T* in intron 9 (rs17409276), *g.21370T* or *p.371D* in exon 10 (rs12711521) and *g.24762C* or *p.S493* in exon 12 (rs1782455) (Table 1). The same SNPs share a smaller, additive effect on the lowest MAp19 levels (less than 100 ng/mL) (Table 1). They constitute the second most common *1B1-h [GA] haplotype, whereas the major alleles at these SNPs define the most frequent *2B2A-i [AG] haplotype. As expected, these haplotypes also present opposite effects on MASP-2 and MAp19 levels (Table 2). Furthermore, the *p.120G* variant and its corresponding *2B2B-l haplotype present a strong additive effect on very low MASP-2 levels (less than 200 ng/mL) (Tables 1 and 2).

**Table 1:** Minor allele frequencies (% ± standard deviation) in the Danish population, leprosy patients and controls, and association with MASP-2 and MAp19 concentration and the disease (adjusted effect ± standard deviation).

	Gene	NG007289.1	NP_006601.2	Danish	Controls	Patients	Lepromatous	Non lepromatous	MASP–2	MASP-2	MAp19	Leprosy
dbSNP	region	Alleles	Aminoacid	N = 582	N = 810	N = 438	N = 262	N = 128	≥600 ng/mL	≤200 ng/mL	≤100 ng/mL	per se
rs7548659	Promoter	*g.4847A>* ***C***	n.a.	20.4	30.2	34.9	35.1	35.9	Dominant:	n.s.	Additive:	Additive:
				±1.7	±1.6	±2.3	±3	±4.3	3.33±0.62		1.28±0.41	1.5±0.54
									P<0.00001		P = 0.0017	P = 0.0053
rs61735600	Exon 3	*g.5557G>* ***A***	*p.R99* ***Q***	0	1	2.5	1.5	4.7	n.a.	n.a.	n.a.	& Dominant:
					±0.3	±0.7	±0.8	±1.9				3.29±1.4
												P = 0.0186
rs72550870	Exon 3	*g.5620A>* ***G***	*p.D120* ***G***	4.5	1.5	2.3	2.3	1.6	n.s.	# Additive:	n.s.	n.s.
				±0.9	±0.4	±0.7	±0.9	±1.1		3.47±1.1		
										P = 0.0016		
rs56392418	Exon 3	*g.5638C>* ***T***	*p.P126* ***L***	0	**0.9**	**4.1**	3.8	3.1	n.a.	n.a.	n.a.	n.s.
					**±0.3**	**±1**	±1.2	±1.5				
												
rs2273344	Intron 4	*g.7164A>* ***G***	n.a.	19.9	16.3	19	18.7	20.3	Dominant:	n.s.	Additive:	n.s.
				±1.7	±1.3	±1.9	±2.4	±3.6	3.04±0.54		1.32±0.41	
									P<0.00001		P = 0.0013	
rs9430347	Intron 5	*g.7441G>* ***A***	n.a.	19.6	16.3	18.5	18.3	19.5	Dominant:	n.s.	Additive:	n.s.
				±1.6	±1.3	±1.9	±2.4	±3.5	3.08±0.55		1.34±0.41	
									P<0.00001		P = 0.001	
rs17409276	Intron 9	*g.21081C>* ***T***	n.a.	14.9	13.3	16.9	16.8	20.3	Dominant:	n.s.	Additive:	n.s.
				±1.5	±1.2	±1.8	±2.3	±3.6	3.58±0.55		1.53±0.42	
									P<0.00001		P = 0.0002	
rs12711521	Exon 10	*g.21370G>* ***T***	*p.Y371* ***D***	17.4	24.3	30.8	31.3	32	Dominant:	n.s.	Additive:	Additive:
				±1.6	±1.5	±2.2	±2.9	±4.1	3.63±0.62		1.33±0.41	1.89±0.55
									P<0.00001		P = 0.0012	P = 0.0005
rs2273346	Exon 10	*g.21389T>* ***C***	*p.V377* ***A***	1.4	4.2	3	3.8	0.8	n.s.	n.s.	n.s.	n.s.
				±0.5	±0.7	±0.8	±1.2	±0.8				
rs12085877	Exon 12	*g.24599G>* ***A***	*p.R439* ***H***	0	**0.6**	**2.5**	2.3	0.8	n.a.	n.a.	n.a.	Dominant:
					**±0.3**	**±0.7**	±0.9	±0.8				3.53±1.5
												P = 0.0184
rs1782455	Exon 12	*g.24762T>* ***C***	*p.S493 = *	16	19.6	27.4	26.7	31.3	Dominant:	n.s.	Additive:	Additive:
				±1.5	±1.4	±2.1	±2.7	±4.1	3.79±0.62		1.44±0.41	2.05±0.56
									P<0.00001		P = 0.0005	P = 0.0002

In bold, italicized: minor allele. n.a.: not applicable; n.s.: not significant. N: number of alleles. In bold: significant differences. # corrected for MAp19 levels, &: not significant with the Fisher’s exact test.

**Table 2:** Haplotype frequencies (% ± standard deviation) in Brazilian leprosy patients and controls, and association with MASP-2 and MAp19 concentration and the disease (adjusted effect ± standard deviation).

Haplotypes	Danish	Patients	Controls	Lepromatous	Non-lepromatous	MASP–2	MASP–2	MAp19	Leprosy	
[Intr4,5]	N = 582	N = 438	N = 810	N = 262	N = 128	≥600 ng/mL	≤200 ng/mL	≤100 ng/mL	per se	
*1A [AG]	0.2±0.2	5.0±1.0	4.0±0.7	4.6±1.3	6.3±2.1	n.s.	n.s.	n.s.	& Additive:	
									2.11±0.04	
									P = 0.036	
*1A [GA]	0.9±0.4	1.4±0.6	1.5±0.4	1.5±0.8	1.6±1.1	n.s.	n.s.	n.s.	n.s.	
*1B1–h [AG]	0.7±0.3	2.5±0.7	1.0±0.3	3.1±1.1	3.1±1.5	n.s.	n.s.	n.s.	n.s.	
*1B1–h [GA]	14.1±1.4	11.9±1.5	11.2±1.1	12.2±2.0	12.5±2.9	Dominant:	n.s.	Additive:	n.s.	
						3.87±0.63		1.6±0.42		
						P<0.00001		P = 0.0001		
*1B2–h [GA]	0	2.5±0.7	1.0±0.3	1.5±0.8	4.7±1.9	n.a.	n.a.	n.a.	& Dominant:	
									3.26±1.4	
									P = 0.0198	
*1C1–l [AG]	0	**1.6±0.6**	**0.2±0.2**	1.5±0.8	2.3±1.3	n.a.	n.a.	n.a.	n.s.	
*1C2–l [AG]	0	**2.5±0.7**	**0.6±0.3**	2.3±0.9	0.8±0.8	n.a.	n.a.	n.a.	Dominant:	
									3.5±1.5	
									P = 0.0194	
*2A1 [AG]	0	0.5±0.3	0.4±0.2	0.8±0.5	0	n.a.	n.a.	n.a.	n.s.	
*2A1 [GA]	0	0	0.1±0.1	0	0	n.a.	n.a.	n.a.	n.s.	
*2A2–l [AG]	1.4±0.5	3.0±0.8	4.2±0.7	3.8±1.2	0.8±0.8	n.s.	n.s.	n.s.	n.s.	
*2B1–i [AG]	0.7±0.3	**2.1±0.7**	**4.4±0.7**	1.5±0.8	3.1±1.5	n.s.	n.s.	n.s.	n.s.	
*2B1–i [GA]	2.6±0.7	2.1±0.7	1.6±0.4	2.3±0.9	0.8±0.8	n.s.	n.s.	n.s.	n.s.	
*2B2A-i [AG]	72.5±1.9	61.6±2.3	67.3±1.7	61.5±3.0	61.7±4.3	Recessive:	n.s.	Additive:	& Additive:	
						–3.24±0.74		–1.9±0.44	–1.11±0.56	
						P<0.00001		P<0.00001	P = 0.046	
*2B2A-i [GA]	2.1±0.6	0.7±0.4	0.9±0.3	0.8±0.5	0	n.s.	n.s.	n.s.	n.s.	
*2B2A-i [GG]	0.3±0.2	0.5±0.3	0	0.4±0.4	0.8±0.8	n.s.	n.s.	n.s.	n.s.	
*2B2B-l [AG]	4.5±0.9	2.3±0.7	1.5±0.4	2.3±0.9	1.6±1.1	n.s.	# Additive:	n.s.	n.s.	
							3.7±1.13			
							P = 0.001			
*2B2A-i.1B1–h[AG]	0.2±0.2	0	0.1±0.1	0	0	n.s.	n.s.	n.s.	n.s.	

N: number of alleles; n.a.: not applicable; n.s.: not significant; #: corrected for MAp19 levels; &: not significant with the Fisher’s exact test. In the phylogenetic *MASP2* nomenclature, small capitalized “h,” “i,” or “l” refer to “high”, “intermediate” or “low” MASP-2 levels in serum, respectively [12]. Within brackets: rs2273344 (intron 4) and rs9430347 (intron 5) variant *in cis* combinations. In bold: significant differences.

### MASP2 Association with Leprosy

**Figure 3:**
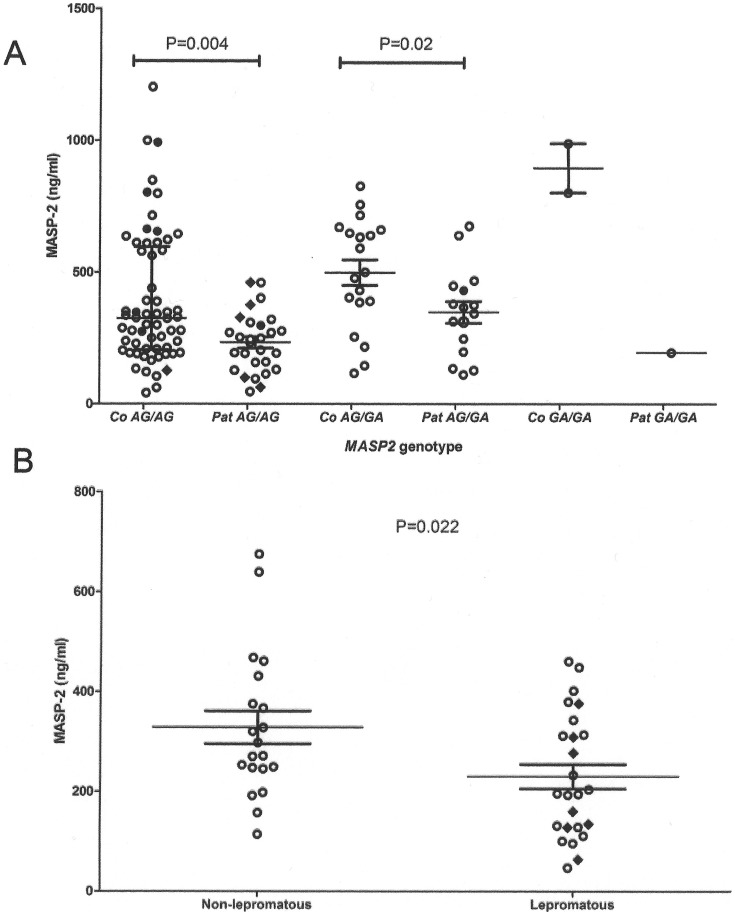
MASP-2 levels in Brazilian controls and leprosy patients. A) Differences between MASP-2 levels of patients and controls according to exon 5 flanking *MASP2* genotypes. Data is shown with medians and interquartile ranges, differences were calculated with Mann-Whitney tests (P_Bf_ = 0.02 between *AG/AG* and P_Bf_ = 0.1 between *AG/GA* groups). Pat: Patients; Co: controls; diamonds: genotypes with *p.126L* (*1C1-l and *1C2-l); closed circles: genotypes with *2B1-i. B) Differences between MASP-2 levels of lepromatous and non-lepromatous patients. Data is shown with means and standard error of the means; differences between groups were calculated with an unpaired t test. Diamonds: genotypes with *1C2-l and/or *2A2-l but without *1B1-h or *1B2-h.

Both leprosy patients and Brazilian controls presented the described profile of MASP-2 levels according to the exon 5 flanking polymorphisms, but patients presented lower MASP-2 levels than controls (medians: 253 vs. 348 ng/mL, respectively, Mann-Whitney P = 0.0012, P_Bf_ = 0.0084) (Figure 3A). These differences were reflected by the haplotype distribution, which was significantly different between patients and controls (P = 0.0011, P_Bf_ = 0.0066). Specifically, the frequency of the *p.126L* variant, which is associated with low MASP-2 levels, was higher in the patients (18/438 or 4.1% vs. 7/810 or 0.9%, P = 0.0002, pFDR = 0.0019, q = 0.0005, OR = 4.92 [95% CI = 2.04–11.86]), as was the frequency of genotypes with *p.126L* (18/219 or 8.2% vs. 6/405 or 1.5%, P = 0.00006, pFDR = 0.0038, q = 0.0005, OR = 5.96 [95% CI = 2.3–15.3]) (Figure 3A). The *p.126L* allelic association was also evident in the Euro-Brazilians (10/352 or 2.8% vs. 3/654 or 0.5%, P = 0.0024, pFDR = q = 0.0012, q = 0.0005, OR = 6.35 [95% CI = 1.7–23.2]). It could actually result from a hitch-hiking effect with the *p.439H* variant, which occurs in absolute linkage disequilibrium with *p.126L* in the *1C2-l haplotype and appears to have a strong dominant effect on the susceptibility to the disease (11/438 or 2.5% vs. 5/810 or 0.6%, P = 0.007, pFDR = 0.0006, q = 0.0005, OR = 4.15 [95% CI = 1.43–12.01]). This association was especially evident for Euro-Brazilian patients (7/352 or 0.2 vs. 1/654 or 0.002, P = 0.0034, pFDR = 0.0007, q = 0.0005, OR = 13.25 [95% CI = 1.62–108.13]). Other variants compounding the *1C2-l haplotype –*g.4847C, p.371D* and *g.24762*–share an additive effect on the susceptibility to the disease (Tables 1 and 2). Genotypes composed solely by the *2B1-i and/or *2B2A-i haplotypes, both associated with intermediate MASP-2 levels (200–600 ng/mL), were found to be protective (224/405 or 55.3% in the controls vs. 93/219 or 42.5% in the patients, P = 0.0014, pFDR = 0.001, q = 0.0005, OR = 0.6 [95% CI = 0.43–0.83] (Figure 3A).

Among the genotypes associated with MASP-2 levels below 400 ng/mL, those with *1C2-l and/or *2A2-l but without *1B1-h or *1B2-h, were more frequent in the lepromatous patients than in those with the other forms (16/131 or 12.2% vs. 1/64 or 1.6%, P = 0.008, pFDR = 0.0006, q = 0.0005, OR = 8.8 [95% CI = 1.1–67.7]). As expected, lepromatous patients presented lower MASP-2 levels than non-lepromatous patients (means ± standard deviation: 229.8±123.8 ng/mL vs. 348±147.3 ng/mL, respectively, unpaired t test P = 0.022, P_Bf_ = 0.044) (Figure 3B).

We performed multivariate analysis to account for any discrepancies in demographic factor distribution and genotype interaction. As expected, genotypes with the *p.126L* and/or *p.439H* variants were not independently associated with the disease, since both can occur in the same haplotype (*1C2-l). Each remained significantly associated with the disease after correction for age, gender, ethnic group and genotypes with the *2B1-i and/or *2B2A-i haplotypes, which did also remain associated with protection against leprosy development (*p.126L* P = 0.001, *p.439H* P = 0.034, *2B1-i and/or *2B2A-i P = 0.049). Significance was lost after the inclusion of genes previously associated with the disease for the same set of patients and controls (only genotypes with FCN2*AGA remained significant-P = 0.026 among genotypes with MBL2*LYPA, FCN1*-542A–144C and MASP2*126L). MASP-2 levels were also associated with the disease after correction for demographic factors (P = 0.001).

## Discussion

Due to extreme difficulties in cultivating *M. leprae in vitro* and its slow growth in animal models, the molecular mechanisms of infection and immune evasion by the bacillus are still poorly known [36]. Nevertheless the importance of complement activation and opsonophagocytosis in mycobacterial monocyte/macrophage invasion has been increasingly underlined by several different polymorphisms of pattern recognition receptors that modulate phagocyte invasion in the host [2]. Among them, genetically-defined low MBL and M-ficolin levels have been found protective by us and by others, against leprosy *per se* and/or the lepromatous disease. In contrast, haplotypes associated with normal L-ficolin levels were protective against clinical leprosy [34] and C4B deficiency (C4B*Q0) increases the risk to pathological immune reactivity in lepromatous disease [37].

Using multiplex sequence-specific amplification for physical *MASP2* haplotyping [12] and the same genotype-phenotype approach that proved to be informative in our previous investigations with MBL and M-ficolin, as well as with MASP-2 in Chagas disease [14], we were able to go deeper in our investigation of lectin pathway components in the susceptibility to leprosy. To this end, we refined our haplotyping strategy to include two common polymorphisms flanking the alternative exon 5, which is exclusive of the truncated MAp19 protein [38]. In fact, the *NG_007289.1:g.7164G* variant in intron 4 and *g.7441A* in intron 5 were as strongly associated with the generation of high MASP-2 and low MAp19 levels, as the formerly reported *g.21081T* polymorphism in intron 9 [12]. The association with the *g.21081T* variant is thus most probably a hitch-hiking effect, since all three intronic variants are strongly linked. In fact, we observed the *g.7164G* variant *in silico* to create a silencer motif which might cause the alternative exon 5 to be skipped in the pre-mRNA splicing process. Interestingly, recombination has shuffled the intron 4 and 5 variants in a manner that they happen to occur in their two common *AG* and *GA* combinations in most of the clade 1 and clade 2 *MASP2* haplotypes with wild-type amino acid sequence (excluding only *p.Y371D*). The modulation of MASP-2/MAp19 levels may thus be of ancient selective advantage, similarly to the splicing variants of endoplasmic reticulum amino peptidase 2 (*ERAP2*), which has a fundamental role in MHC class I presentation [39]. This balance of alternative mRNA processing may differ for different cell lineages, since it is known that MAp19 is mainly expressed in Kupffer cells, whereas MASP-2 is mainly synthesized in hepatocytes [38].

Surprisingly, we found that genetically-defined low MASP-2 levels do not mimic the protective association found between MBL and M-ficolin deficiencies and leprosy. Instead, low MASP-2 levels generated by *p.126L*, *p.377A* and/or *p.439H* combined with the exon 5 flanking *AG* variants were associated with susceptibility to leprosy *per se* as well as with lepromatous disease. Among these variants, *p.439H* is the only one known to hinder complement activation. This mutation disrupts the activation peptide. In contrast to *p.120G,* which is also known to cause MASP-2 deficiency [8]–[10], the resulting protein binds to MBL. Nevertheless it cannot autoactivate [10]. Thus, in addition to being unable to activate complement, *p.439H* probably functions as a “sink” for MBL, reducing binding opportunities with full-working MASP-2 homodimers in heterozygotes. This could also be the underlying reason for its dominant effect on the susceptibility to the disease. Importantly, the distribution of *p.120G* did not differ between patients and controls, as well as between lepromatous and non-lepromatous patients. One might argue that, beside functional differences, the contrasting results between *p.439H* and *p.120G* could be the result of population substructure, since *p.439H* are most probably of African, and *p.120G* of European origin [9]–[11]. Nevertheless the association of the *1C2-l haplotype (harboring both *p.126L* and *p.439H*) with leprosy *per se* was especially evident after the exclusion of Afro-Brazilian patients, which present at least 40% of sub-Saharan African admixture [23], [24], whereas the frequencies of *p.120G* in patients and controls remained similar.

Both leprosy and malaria are intracellular infections associated with the same MBL and MASP-2 variants that disturb the lectin pathway of complement activation, nevertheless with opposite effects on the disease susceptibility. In sharp contrast with leprosy, where susceptibility to the disease is associated with haplotypes generating high MBL levels [35] and low MASP-2 levels (this study), low MBL levels generated by the MBL2*LYQC haplotype increase susceptibility to malaria [16], [40]–[42], whereas low MASP-2 levels generated by the *p.439H* variant are protective [16]. These opposite effects cannot be explained solely on the basis of disturbed complement activation, since MBL and MASP-2 are known to act synergistically in this process. A possible explanation could be the ability of MASPs to block the interaction site of MBL with the CD91/calreticulin complex on the macrophage cell membrane, thereby preventing MBL-driven phagocytosis [43]. If this mechanism is operating in mycobacterial cell invasion, low MASP-2 levels would be insufficient to avoid subsequent infection. Surely this hypothesis needs to be tested in a functional setting, maybe using the recently achieved bioengineered BCG bacillus [36]. Otherwise in malaria, the ingestion of hemozoin could be beneficial to control for dyserithropoiesis [44]. It should also be kept in mind that MASP-2 is a pleiotropic molecule, being involved in the coagulation cascade through the cleavage of prothrombin, generating cross-linked fibrin covalently bound on bacterial surfaces [45], [46]. Disruption of this pathway could modulate susceptibility to infections, as well. In addition to low MASP-2, high MAp19 levels could play a role in leprosy pathogenesis, but the strong association with *p.439H*, which does not occur in the truncated protein, disfavors an important role for it. Finally, it should be kept in mind that associations of genes of the lectin pathway of complement with the disease are not independent of each other. Multivariate analysis predicted a more important role for the FCN2*AGA haplotype [34], known to be associated with normal L-ficolin levels, but whose functional relevance awaits further investigation.

### Conclusion

The results lead us to suggest that, in contrast to MBL, low MASP-2 levels increase the susceptibility to leprosy in general and to lepromatous leprosy in particular. *MASP2* genotypes and MASP-2 levels might be used as biomarkers to predict disease progression in leprosy.

## Acknowledgments

The subjects of this investigation were informed about the aims of the study and their consent to participate is gratefully acknowledged. We are also thankful to the medical staff of the Hospital de Clínicas of the Federal University of Paraná and of the Sanitary and Dermatologic Hospital of Paraná for patient recruitment, to the staff of the Laboratório de Imunopatologia Molecular in Curitiba and Rudi Steffensen of the Department of Clinical Immunology in Aalborg for DNA extraction, and for Flávia R. Nass and Renato M. Nisihara for excellent technical assistance in performing ELISA assays.
